# Non-invasive Production of Multi-Compartmental Biodegradable Polymer Microneedles for Controlled Intradermal Drug Release of Labile Molecules

**DOI:** 10.3389/fbioe.2019.00296

**Published:** 2019-11-08

**Authors:** Mario Battisti, Raffaele Vecchione, Costantino Casale, Fabrizio A. Pennacchio, Vincenzo Lettera, Rezvan Jamaledin, Martina Profeta, Concetta Di Natale, Giorgia Imparato, Francesco Urciuolo, Paolo Antonio Netti

**Affiliations:** ^1^Center for Advanced Biomaterials for Health Care (CABHC), Istituto Italiano di Tecnologia, Naples, Italy; ^2^Interdisciplinary Research Centre on Biomaterials (CRIB), University of Naples Federico II, Naples, Italy; ^3^Biopox Srl, Naples, Italy; ^4^Department of Chemical Materials and Industrial Production (DICMAPI), University of Naples Federico II, Naples, Italy

**Keywords:** polymer microneedles, multi-compartmental, enzyme, controlled release, skin model

## Abstract

Transdermal drug delivery represents an appealing alternative to conventional drug administration systems. In fact, due to their high patient compliance, the development of dissolvable and biodegradable polymer microneedles has recently attracted great attention. Although stamp-based procedures guarantee high tip resolution and reproducibility, they have long processing times, low levels of system engineering, are a source of possible contaminants, and thermo-sensitive drugs cannot be used in conjunction with them. In this work, a novel stamp-based microneedle fabrication method is proposed. It provides a rapid room-temperature production of multi-compartmental biodegradable polymeric microneedles for controlled intradermal drug release. Solvent casting was carried out for only a few minutes and produced a short dissolvable tip made of polyvinylpyrrolidone (PVP). The rest of the stamp was then filled with degradable poly(lactide-co-glycolide) (PLGA) microparticles (μPs) quickly compacted with a vapor-assisted plasticization. The outcome was an array of microneedles with tunable release. The ability of the resulting microneedles to indent was assessed using pig cadaver skin. Controlled intradermal delivery was demonstrated by loading both the tip and the body of the microneedles with model therapeutics; POXA1b laccase from *Pleurotus ostreatus* is a commercial enzyme used for the whitening of skin spots. The action and indentation of the enzyme-loaded microneedle action were assessed in an *in vitro* skin model and this highlighted their ability to control the kinetic release of the encapsulated compound.

**Graphical Abstract:**
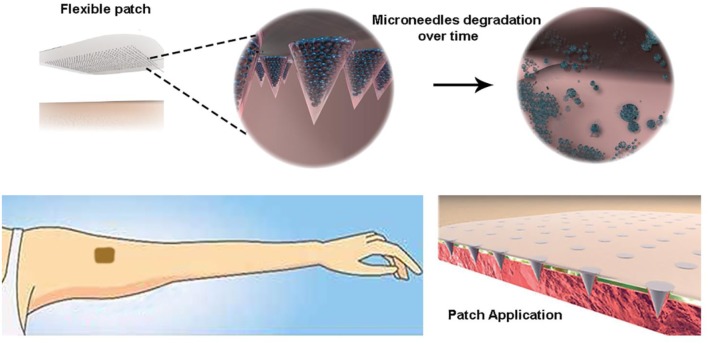
Highly controlled intradermal delivery can be performed with multi-compartmental polymer microneedles that are able to indent the skin and be implanted in it with a fast dissolving tip and a slow degradable body made of porous PLGA μPs.

## Introduction

Transdermal drug delivery is an attractive alternative to parenteral administration. It bypasses the hepatic first-pass metabolism, preventing intestinal degradation and reducing systemic drug exposure (Kumar and Philip, 2007; Chu et al., 2010; Nguyen et al., 2018). However, classic transdermal delivery systems are severely limited by the inability of the vast majority of drugs to cross the skin barrier imposed by the stratum corneum, the outermost lipophilic layer of the skin (thickness of 10–30 μm), which only allows the transport of molecules of fewer than 400 Da (Schaefer et al., 1997, 2011; Naik et al., 2000; Bouwstra and Ponec, 2006).

Several techniques, such as microdermabrasion, laser ablation, radiofrequency, and thermal abrasion, are used to enhance skin permeability (Kumar and Philip, 2007). However, such techniques can seriously damage the skin, are painful for patients, and require both skin treatment and agent application.

These limitations can be overcome using needles of micrometer dimensions, namely microneedles, which penetrate the skin and then release their cargo (Bronaugh and Maibach, 1999; Joshi and Raje, 2002; Prausnitz et al., 2004). In fact, when inserted into the skin, they create microchannels that enable a broad range of therapeutic agents to be delivered, such as drugs, vaccines, enzymes, proteins, and peptides (Park et al., 2006; Jiang et al., 2007; Sullivan et al., 2010; Kim et al., 2012; Matsuo et al., 2012; Tsioris et al., 2012; Hiraishi et al., 2013; Edens et al., 2015a,b; Lee et al., 2015; Arya et al., 2016; Qiu et al., 2016; Ruggiero et al., 2018), without inducing serious skin damage, irritation, or infection (Nguyen and Banga, 2017). They are also painless because they do not stimulate the dermal nerves (Donnelly et al., 2010). Microneedle-based technology has also attracted the attention of the cosmetic industry for skin aging, acne, photodamage, and hyperpigmentation treatment (Preetha and Karthika, 2009).

One of the commonest and most reliable fabrication methods for polymeric microneedle preparation is the stamp-based technique (Raja et al., 2013; Yang et al., 2015). However, microneedles are commonly made of water-soluble materials, such as polymers and sugars, that will dissolve into the skin a few minutes after insertion (Park et al., 2005; Kim et al., 2012) or of biodegradable polymers that need to remain embedded in the skin for several days to promote a prolonged release (Park et al., 2006).

To overcome the long application times of the biodegradable polymers, Li et al. proposed a microneedle patch with rapidly separable biodegradable PLGA needles for the continuous release of a contraceptive hormone. The fabrication method consists of the casting step and the formation of a bubble at the base of the cone that rapidly separates the needles from the patch. Despite the interesting results obtained in terms of microneedle penetration in the skin, microneedle detachment from the patch, and therapeutic agent delivery, this method does not allow for a tunable drug release of the compounds over time (Li et al., 2019).

A spray-based method has recently been introduced to obtain controlled biphasic release (Park et al., 2019). Microneedles are detached from the stamp after overnight dehydration. A slow release from the biodegradable compartment is not easily tunable. Previously, to overcome the limitations related to controlling the delivery rate, a new approach had been proposed by Irvine (DeMuth et al., 2013) that combined poly(acrylic acid) (PAA), as a fast dissolvable polymer matrix, and PLGA μPs for sustained release. However, despite the clear improvements obtained, this method remains time consuming (at least 48 h). Apart from being expensive, long fabrication processes can promote the unloading of hydrophilic cargos from the μPs to the PAA matrix as well as the loss of moisture-sensitive drugs during the long drying step.

In order to overcome these drawbacks, we have developed a novel and fast stamp-based method for fabricating multi-compartmental polymeric microneedles with tunable drug delivery properties.

The first step in the production of microneedles was the fabrication of a master using a 2 photon polymerization (2PP) technique that ensures a tip curvature radius smaller than 20 μm (Pennacchio et al., 2017, 2018). Then, after replicating PDMS stamps, multi-compartmental microneedles were fabricated. First, the cavities of the stamp were spin-coated with a hydrophilic polymer, the PVP, to obtain the fast-dissolvable tips, according to a recently proposed method (Vecchione et al., 2016). PVP is mechanically strong because of its chemical backbone, which contains rigid rings, and it has been widely used in the production of polymer microneedles (Lopérgolo et al., 2003). PVP is also water soluble, facilitating the rapid dissolution of the microneedles when inserted into the skin. Once dissolved in the body, PVP can be safely cleared within a few days (Kodaira et al., 2004). The cavities were then filled with porous PLGA μPs and assembled by a mild-softening method, creating the body of the microneedle, which is devoted to the prolonged release of the cargo. PLGA is one of the most successfully used biodegradable polymers approved by the Food and Drug Administration (FDA) and the European Medicine Agency (EMA) due to its biodegradability and biocompatibility (Bobo et al., 2016).

It is thus possible to fabricate multi-compartmental microneedles with tunable drug release properties that combine the fast release of the tip with the prolonged release of the cargo from the μPs as well as the possible co-delivery of two molecules.

Interestingly, the whole process is carried out at RT, thus allowing the encapsulation of thermo-sensitive molecules. We assessed the compliance of the method by monitoring the activity of an enzyme, namely POXA1b laccase from *Pleorutus ostreatus*, which was encapsulated both in the tip and the microparticles.

The performance of the microneedles was highlighted *in vitro* by assessing their indentation capacity in a pig cadaver skin model together with the enzyme diffusion and activity in an advanced *in vitro* human skin equivalent model.

## Materials and Methods

### Materials

Poly (lactic-co-glycolic acid) 50:50 (PLGA RESOMER® RG 504H, 38,000–54,000 Dalton), was purchased from Boeringer Ingelheim. Polyvinylpyrrolidone (PVP 856568 Mw 55 KDa), Pluronic® F-68, dimethyl carbonate (DMC, D152927), dichloromethane (DCM) sodium acetate anhydrous, sulphorhodamine B (Rhod) (SulphoRh6G, S470899), fluorescein isothiocyanate isomer I (FITC), and 2,2′-azino-bis(3-ethylbenzothiazoline-6-sulfonic acid) diammonium salt (ABTS) were purchased from Sigma Aldrich. NOA 61 glue was purchased from Norland Optical Adhesive. Poly (dimethyl-siloxane) (PDMS) was provided by Sylgard® (184 Silicone Elastomer Kit, Dow Corning). Poly(methyl methacrylate) (PMMA) was purchased from GoodFellow. The medical tape came from 3 M. Pure recombinant POXA1b (rPOXA1b) laccase enzyme was provided by Biopox srl. IP-DIP negative tone photoresist was purchased from Nanoscribe GmbH. Bidistilled water was pretreated with a Milli-Q R Plus System (Millipore Corporation, Bedford, USA). The MPatch™ Mini Applicator was purchased from Micropoint Technologies Pte Ltd.

### Laccase-Atto 647 Conjugation

Laccase (Biopox, Naples, Italy) was covalently conjugated to Atto-647 NHs by ATTO-TEC GmbHprocedure (https://www.atto-tec.com/fileadmin/user_upload/Katalog_Flyer_Support/Procedures.pdf). To summarize, a solution of 2 mg/mL of the enzyme (Carbonate buffer, pH 8.5) was mixed with a 10-fold molar excess of reactive dye for 1 h at 25°C under magnetic stirring. Unbound dye was removed using a 5 kDa molecular weight cutoff Amicon Ultra-4 centrifuge filter. The correct enzyme-dye conjugation was validated by the UV-vis technique.

### Preparation of PLGA Microparticles

PLGA μPs were prepared by double emulsion solvent evaporation (De Alteriis et al., 2015). Briefly, the aqueous phase, composed of 100 μl of a 0.55 mg ml^−1^ sulforhodamine B aqueous solution or 70 U of pure recombinant POXA1b water solution, was homogenized (Ultra-turrax, IKA T-25 ULTRA-TURRAX Digital High-Speed Homogenizer Systems) for 30 s at 15,000 rpm with 1 ml of a DCM solution composed of 83 mg ml^−1^ of PLGA and 17 mg ml^−1^ of Pluronic F68. The first emulsion was immediately poured into 10 ml of 2% (w/v) aqueous PVA solution and further homogenized for 1 min at 20,000 rpm. The second emulsion was immediately poured into 40 ml of water under mechanical stirring for 3 h at 450 rpm with a paddle stirrer for complete solvent diffusion and evaporation.

The same procedure was also used for Laccase-Atto647-loaded μPs. Briefly, a concentration of 8.4 μg/mL of protein was encapsulated, and, after washing, the microparticles were characterized by fluorescent microscopy (Olympus IX73P1F fluorescence microscope Tokyo, Japan) in order to evaluate the signal of the enzyme inside porous structures. Fluorescence images were acquired using an HCX IRAPO L 40×/0.95 water objective with a 633 nm laser as the excitation source. In order to maintain the enzyme activity, the laccase-loaded microparticles were entirely prepared under refrigeration on ice. The μPs suspension was then centrifuged three times for 10 min at 4°C and 10,000 rpm (AvantiTM J-25, Beckman, USA) for washing. Finally, μPs were lyophilized overnight (Heto PowerDry PL6000 Freeze Dryer, Thermo Electron Corp., USA; −50°C, 0.73 hPa).

### Morphological Characterization of the Microparticles

Microparticle morphology was investigated through a scanning electron microscope (SEM). SEM samples were prepared, depositing 50 μg of μPs on a cover slip mounted on a standard SEM pin stub. The samples were gold-sputtered (10 nm thickness) with a HR208 Cressington sputter coater and analyzed by FESEM ULTRA-PLUS (Zeiss) at 5 kV with the SE2 detector. μPs mean size was determined by static light scattering (Mastersizer 2000, Malvern Instruments, Malvern, UK) on a 0.4 mg ml^−1^ μPs suspension in water.

### Laccase-Atto647 Entrapment Efficiency (%η) Within the μPs

The %η Laccase-Atto 647 μPs was measured by an extraction method as previously reported (Kirby et al., 2011). Briefly, 10 mg of Laccase-Atto647 μPs were incubated in a solution of 50% dimethyl sulphoxide (DMSO)/0.5% Sodium dodecyl sulfate (SDS)/0.1 M Sodium hydroxide (NaOH) for 1 h, at 25°C. The final solution was then analyzed by fluorescence, using an EnSpire® Multimode Plate Reader and following the signal of Laccase-Atto647 at 663 nm. The content of the enzyme loaded was obtained using a titration curve of the free Laccase-Atto 647 conducted at the same time and under the same conditions (Figure S1A).

### Fabrication of Master and Flexible Support

The microcone master was fabricated by means of 2-photon polymerization (2PP) using the Nanoscribe Photonic Professional GT system (Nanoscribe GmbH). The Nanoscribe system uses a 780 nm Ti-Sapphire laser emitting ≈100 fs pulses at 80 MHz with a maximum power of 150 mW and equipped with a 63×, 1.4 NA oil immersion objective. The substrate was placed in a holder that fitted into a piezoelectric x/y/z stage. A galvo scanner determined the laser trajectories.

The master was fabricated by processing the IP-DIP negative tone photoresist (Nanoscribe GmbH). To speed up the process, a galvo stage was used, which is almost 100 times faster than the basic piezo mode setup. The basic master was fabricated with a conical shape and with a microneedle height of 600 μm and base diameter of 300 μm. To enhance the photoresist adhesion, an oxygen plasma treatment (1 min) was performed. A layer of IP-DIP was spin-coated onto the glass substrate (5,500 rpm for 30 s) and photo-polymerized by UV lamp exposure (16 mW/cm^2^ for 4 h). A drop of photoresist was then dispensed onto the glass substrate and fabricated with the Nanoscribe to directly produce the microcone master.

The master produced by 2PP was put under a UV lamp (3 h) to induce microcone hardening before use. The master structure was then replicated by pouring a solution of liquid PDMS precursor and its curing agent (10:1 w/w) onto it. This was then put under vacuum to remove entrapped air bubbles and cured in an oven at 70°C for 1 h. After curing, the PDMS was peeled off and attached onto double-sided adhesive tape on a glass slide; then, NOA 60 was poured onto it and put under vacuum to remove the bubbles for about 1 h. NOA 60 is a clear, colorless, liquid photosensitive polymer that is cured when exposed to ultraviolet light in the wavelength range of 350–380 nm (www.norlandprod.com). The curing time depends on the thickness and on the energy of the ultraviolet light. In our case, the wavelength light was 365 nm and the optimized exposure time was 3 h. Finally, the PDMS negative stamp was removed and the NOA positive mold was obtained. The NOA positive mold was attached onto a petri dish or glass slide with double-sided adhesive tape. Finally, the PDMS precursor was poured onto the NOA positive mold, cured at 70°C for 1 h, and then peeled off from the NOA to obtain PDMS stamps. The master produced can thus be used many times without being damaged.

### Fabrication of the Microneedles

Microneedles tips were produced using the hydrophilic polymer PVP. Briefly, 200 μL of 25% (w/v) PVP aqueous solution was poured onto the PDMS stamp and then degassed and spin-coated (3,000 rpm, 1 min) (Laurell Technologies, WS-650-23NPP Spin Coater) to remove the excess. The PDMS stamp cavities were filled with the μPs with the aid of an optical stereomicroscope (Olympus, SZX16 double objective). For each stamp, several arrays of PMMA pillars were produced using a micromilling machine (CNC micromill, Minitech MiniMill 3/Pro, Minitech Machinery Corporation, Georgia, USA) to press the μPs into them. The μPs loaded in the stamps were then plasticized and induced with a solvent mixture made up of 4 ml of ethanol and 0.5 ml of DMC.

The temperature and the pressure were set at 25.3°C and 0.15 bar, respectively (Kodaira et al., 2004) while the time of exposure was 6 min. Microneedles were extracted from the stamps using a harvesting layer, produced as follows: the medical tape (3 M) was placed on a PMMA slice with the aid of double-sided adhesive tape and 200 μl of 25% (w/v) PVP aqueous solution was poured onto the adhesive medical tape (3 M) surface, dried out at 40°C for 2 h, and finally plasticized for 11 min under the previously reported conditions. The harvesting layer was then placed upon the stamp. After 3 h of drying at RT, the microneedles were extracted.

Depending on the type of analysis, FITC-loaded or enzyme-loaded microneedle tips were also produced by adding 0.1 mg ml^−1^ of FITC or 70 U of enzyme into the 25% (w/v) PVP aqueous solution.

### Characterization of the Microneedles

#### Optical Microscopy

After fabrication, the microneedles were analyzed by a stereomicroscope (Olympus, SZX16 double objective) to investigate the sharpness of the tips and the pattern distribution.

#### Scanning Electron Microscopy

Microneedle morphology was investigated through a scanning electron microscope (SEM). SEM samples were prepared by attaching the microneedle patch onto a cover slip mounted on a standard SEM pin stub. The samples were gold-sputtered with a sputter coater (15 nm thickness) and analyzed by FESEM ULTRA-PLUS (Zeiss) at 10–20 kV with the SE2 detector.

#### Fluorescent Microscopy

Fluorescent microscopy analyses were performed using FITC-loaded tips and Rhod-loaded μPs. Sample morphology was investigated using a confocal microscope (Leica Microsystems TCS SP5 II, Germany) with a 20× air objective. Images were acquired with a resolution of 1,024 × 1,024 pixels.

### Slicing of Microneedles and Microparticles

The internal morphological structure of both the microneedles and μPs was investigated according to a previously reported procedure (Kodaira et al., 2004). First, a 2 mm thickness PDMS base layer was produced and cured at 80°C for 30 min. After cooling, the microneedles or μPs were deposited on it and another 2 mm thickness PDMS layer was used to cover the microneedles and μPs. The PDMS was cured for 24 h, at RT, in order to preserve the state of the μPs and microneedles incorporated in it. Finally, the solid PDMS block was frozen in liquid nitrogen (−196°C) and sectioned using a Leica CryoUltra Microtome EM-FC7-UC7. A total of five μm slices were deposited onto a 12 mm round glass cover slip for the confocal analysis and then mounted on a standard SEM pin stub for SEM imaging.

### Image Analysis of Microneedles and Microparticle Slices

To check the microstructure of the porous microneedles and μPs, sample slices were analyzed using a field emission SEM (FESEM ULTRA-PLUS, Zeiss). The samples were gold-sputtered with a sputter coater (15 nm thickness) and analyzed by SEM at 10 kV with the SE2 detector.

To investigate the distribution of the chromophores inside the microneedles, slices of fluorescent microneedles and μPs were then imaged by a confocal microscope (Leica Microsystems TCS SP5 II, Germany) with a 20× air objective. Images were acquired with a resolution of 1,024 × 1,024 pixels. A 3D reconstruction from the z-stack slices was made using LAS AF software.

### *In vitro* Release of Laccase-Atto647 From μPs Inside the Microneedles

Three patches of microneedles with Laccase-Atto647 μPs were suspended in 1.5 mL of phosphate buffered saline (PBS) pH 7.1 and incubated at 37°C under continuous stirring at 250 rpm. At different time periods (from 30 min to 24 h), 1 mL of PBS was removed and analyzed by fluorescence assay as previously reported. The patches were refreshed with the same amount of PBS. Calibration curves of free enzymes were prepared in PBS as previously described (Figure S1B).

### Microneedle Indentation Test in Cadaver Pig Skin

Microneedle arrays of 600 μm height were inserted into full thickness cadaver pig skin without the subcutaneous fat layer. The skin was shaved with depilatory cream and washed in a phosphate buffered saline (PBS) solution and placed on absorbent paper for a few minutes to eliminate excess water. Two different indentation tests were carried out with an MPatch™ applicator system. This applicator is engineered to apply microneedle patches effectively by ensuring a 100% penetration rate. The penetration speed and force are optimized to provide repeatable and reproducible results (www.micropoint-tech.com).

### Histological Analysis

The microneedle patch was removed after indentation and the skin was fixed in a solution of 10% neutral buffered formalin (Bio-Optica) for 24 h, dehydrated in an incremental series of ethanol (75, 85, 95, 100, and 100% again, each step performed for 30 min at RT), treated with two series of xylene (Sigma Aldrich) for 30 min, and then embedded in paraffin (Bio-Optica). Samples were then sectioned with a thickness of 3 μm and stained with hematoxylin and eosin (Bio-Optica), and finally the sections were mounted with Histomount Mounting Solution (INVITROGEN) on coverslips, and the morphological features of constructs were observed by optical microscope (BX53; Olympus).

### Enzyme Analysis

Laccase activity was determined by the ABTS oxidation method (Lettera et al., 2010). ABTS is oxidized by laccase to the most stable and preferred state of the cation radical. The concentration of the cation radical responsible for the intense blue-green color can be correlated to the enzyme activity and is read at 420 nm (Majcherczyk et al., 1998). The assay mixture contained 2 mM ABTS, 0.1 M sodium acetate (pH 3.0), and the enzymatic solution. In the case of enzymes encapsulated in μPs, 5 mg of μPs were dissolved in 200 μl of DMC for 1 min; then, 5 μl of DMC dissolved μPs solution were assayed in a final volume of 1 ml. Oxidation of ABTS was monitored by determining the increase in A_420_ (ε_420_, 3.6 × 10^4^ M^−1^·cm^−1^). Absorbance was read at 420 nm in a spectrophotometer against a suitable blank. One unit was defined as the amount of laccase that oxidized 1 μmol of ABTS substrate per min.

### Comparison of Spatial Profile of the ABTS Oxidation Product Concentration at Different Time Points After Microneedle Array Indentation in the Full-Thickness Skin Model

Endogenous-Human Skin Equivalent (Endo-HSE) (Casale et al., 2016, 2018) was used as the assay platform to evaluate the diffusion profile of the oxidation product formed by the reaction of ABTS soaked in Endo-HSE with the Laccase encapsulated in the array of microneedles. Endo-HSE was soaked in the ABTS solution (11 mg/ml in sodium citrate) for 30 min at RT. In addition, Endo-HSE was kept on absorbent paper to remove excess solution and indented by microneedles in both configurations. Medical tape was used to maintain the microneedles and allow indentation. The medical tape was removed 1 min after the indention had been performed. The enzyme was loaded in the hydrophilic tip (configuration 1) or in the porous μPs (configuration 2), and its activity was assessed *in vitro*.

Observations of the epidermal side of Endo-HSE after indentation in both configurations were performed under a stereomicroscope. Images were recorded at different time points with the same magnification and in the same lightness conditions. The time sampling for each photo was 1, 30 min, 24, and 48 h after the indentation and medical tape removal. The images obtained were then analyzed using image analysis software (Image J®) to calculate the spatial profile of the ABTS oxidation product concentration at different time points. Briefly, each image was converted into 8-bit format and after the lookup table had been inverted. The diameter of the circular spatial profile of the ABTS oxidation product shown by the blue dark stain was subsequently measured three times. The intensity of the brightness pixel scale along the diffusion radius tracked for three time points were shown in the graphs. Three Endo-HSEs for each microneedle configuration were used for the diffusion tests. A total of five images for each sample (about eight microneedles per image) and for each time point were used for the analysis.

## Results and Discussion

### Microparticles

**Figure 1:**
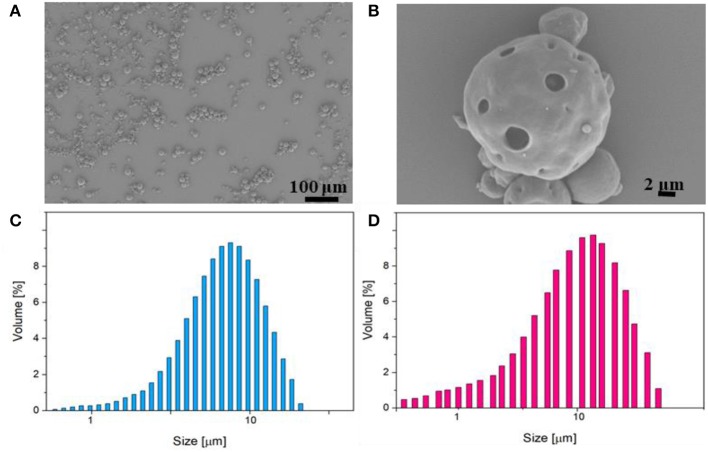
**(A,B)** SEM images of enzyme-loaded μPs at different magnifications. Particle size distribution analysis of PLGA μPs performed by Mastersizer 3000. **(C)** Sulphorhodamine-loaded μPs. **(D)** Laccase-loaded μPs.

PLGA μPs were chosen to produce the body of the multi-compartmental and biodegradable polymeric microneedles. The μPs were produced by means of double emulsion in order to obtain porous microstructures that ensure the encapsulation of hydrophilic molecules and show a kinetic degradation for extended release over time. Microparticle morphology was assessed using SEM microscopy. The images show that μPs have a homogeneous and spherical shape (Figure 1A) as well as a porous surface (Figure 1B). Particle size was analyzed with a Malvern mastersizer. The data show that microparticles have a narrow size distribution with a slightly higher mean diameter in the case of enzyme encapsulated μPs (from ~8 to 10 μm) (Figures 1C,D).

### Master and Stamp

The microcones of the master were individually prepared in a serial production by 2PP onto a glass substrate. In order to improve the material-substrate adhesion of the microcones, the substrate was first treated with oxygen plasma and then a thin layer of photoresist was spin coated and cured on it. Finally, another layer of uncured photoresist was dispensed and processed by 2PP according to the defined 3D layout. In order to optimize the master fabrication time, only the external shell of the microcones was photopolymerized by 2PP, whereas their body was one-shot cured under a UV lamp.

A master of the microcones was produced with 300 μm base diameter and 600 μm height extended onto an area of 1 cm^2^. A maximum density of 256 microneedles *per* cm^2^ was chosen in order to avoid any “bend-of-nails” effect. In fact, if the tips bend when designing arrays of microneedles, this has a negative impact on the microneedles' ability to penetrate the skin as the insertion force would be distributed among too many microneedles so that none would be able to penetrate the skin (Yan et al., 2010).

Because of the fragility of the master material, the polydimethylsiloxane (PDMS) stamp was not used directly. In fact, in order to avoid master breakage, a less fragile master was fabricated using Norland Optical Adhesive (NOA) 60. Therefore, starting from the original positive master, a negative PDMS stamp was replicated on it first and then a positive replica was obtained using NOA. This stamp was then used as a final master, according to the procedure reported in the Materials and Methods section.

**Figure 2:**
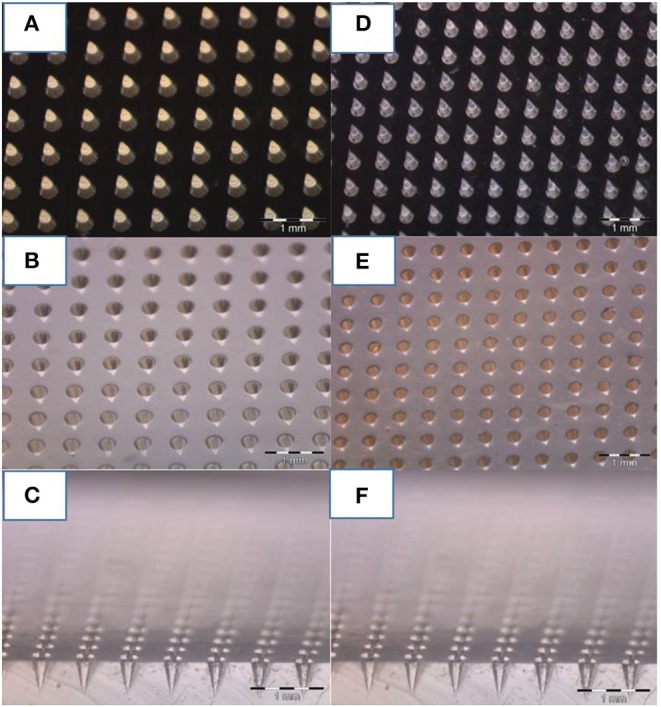
Optical image of microneedles molded at various steps. **(A)** Master of 600 um of height and 300 um of bases of microneedles fabricated by 2PP, **(B)** PDMS stamp replicated on the master, and **(C)** cross-section of the PDMS mold. **(D)** NOA master replicated on the PDMS stamp, and **(E)** final PDMS stamp replicated on it **(F)** including a cross-section. A comparison of photos **(C,F)** reveal how, despite the various stages of replication, the needles keep tips with the same curvature radius.

Optical images of the master produced by 2PP, the PDMS replica, the NOA master, and the final PDMS stamp are shown in Figure 2. The high quality of the PDMS stamps, achieved through replication from the starting master and from the NOA master, is highlighted in Figures 2C,E, which show sections of the tips belonging to the PDMS stamps.

### The Fabrication of Multi-Compartmental Polymeric Microneedles

**Figure 3:**
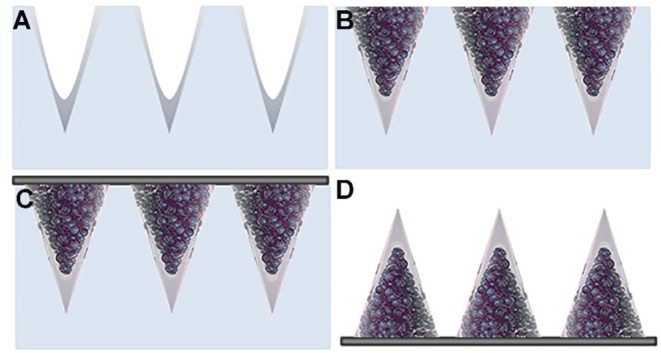
Schematic view of the multi-compartmental microneedle array. PDMS stamp with **(A)** PVP deposited by spin coating and with **(B)** PLGA μPs compacted and sintered. **(C)** Application of the harvesting layer on the stamp. **(D)** Microneedle array extracted from the stamp.

The initial stage of the fabrication of the multi-compartmental microneedles consisted of pouring an aqueous PVP solution onto the PDMS stamp, which was then degassed and spin coated in order to spread out the excess solution from the stamp and allow a rapid evaporation of the residual solvent. The tips of the microneedles were thus produced (Figure 3A). Depending on the experimental needs, FITC- or enzyme-loaded tips were produced. PVP was chosen as the structural material for the fabrication of the microneedle tips because of its processability in an aqueous environment and at RT. These process conditions facilitate the encapsulation of several drugs, which are known to be often denaturized by the interaction with an organic solvent and/or by the presence of high temperatures. Subsequently, stamp cavities were filled with PLGA μPs according to the procedure reported in the experimental section.

The stamp was then exposed to a solvent/non-solvent vapor mixture in order to carry out a mild plasticization for assembling; the μPs thus create the body of the microneedles while keeping the microstructure of the starting μPs (Figure 3B). This mild softening method (i.e., at room temperature using a solvent/non-solvent vapor mixture) was developed in one of our previous works for the mild deformation of single μPs located in the cavities of a PDMS stamp (Kodaira et al., 2004). Here, this patented method was applied for the first time to multiple μPs in order to promote their assembly by gentle sintering within the cavities of the stamp. Sharp microneedles were thus produced without needing to use the strong plasticization of single μPs (Kodaira et al., 2004). This approach preserves not only the internal microstructure of the μPs, but also the properties of the encapsulated drug, also allowing the use of thermo-labile cargo.

Finally, polymer microneedles were extracted from the stamp with the aid of a 3 M medical tape coated with PVP according to the procedure reported in the experimental section (Figure 3C). Figure 3D shows the double compartment microneedles peeled off from the PDMS stamp.

To the best of our knowledge, the most similar procedure reported for the fabrication of biodegradable microneedles is the one published by Irvine et al., who manufactured arrays of microneedles composed of drug-loaded PLGA μPs with a supporting and rapidly water-soluble PAA matrix (Park et al., 2019). The main limitation to this method is the assembly procedure. In fact, the dehydration of the matrix takes 48 h, which, apart from being a time-consuming and expensive step, can lead to the unloading of hydrophilic cargo from the μPs and its consequent diffusion into the external polymer matrix. Moreover, sensitive drugs may lose their activity because of the surrounding moisture.

On the other hand, a key advantage of our method is that it allows the polymer solution to dehydrate in just a few minutes. In addition, it is possible to work with particle sizes of up to 10 μm with a wider realm of possible porosities and therefore higher tunability of the kinetic release.

In the present work, the multi-compartmental feature of the proposed microneedles was obtained with a rapid protocol that provides a tip made of a hydrophilic polymer, namely PVP, and a body made of porous PLGA μPs, both embedding the enzyme. This facilitates the rapid release of the enzyme from the tips, followed by a prolonged release promoted by the μPs. The need for a combined release from multi-compartmental microneedles has also recently been addressed with a combination of PVP and PLGA deposited by spray coating (Park et al., 2019). In this case, the time to detach the microneedles from the stamp is <48 h but it still requires overnight dehydration. In addition, the biodegradable PLGA matrix is deposited by spray coating so the microstructure is not easy to tune, nor is the kinetic release from this compartment. Conversely, porous PLGA μPs can be provided with several microstructures that can promote a huge variety of kinetic releases.

### Morphological Microneedle Characterization

**Figure 4:**
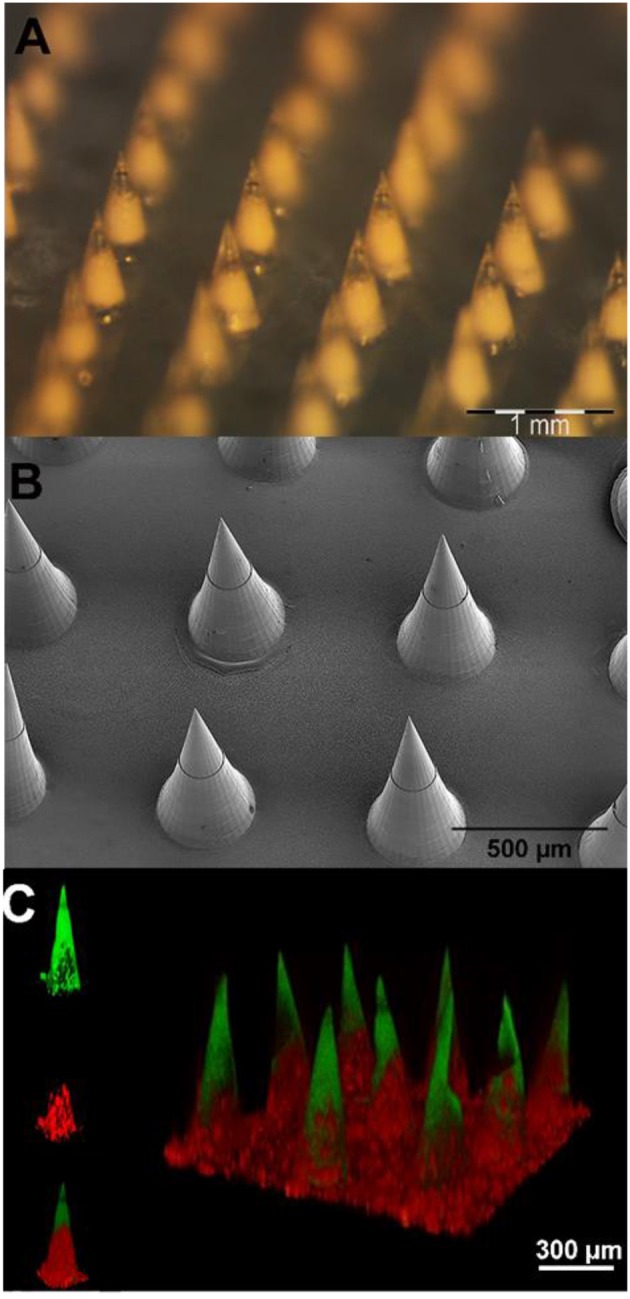
**(A)** Stereomicroscope micrographs of microparticle-loaded microneedle patches. **(B)** Scanning electron microscopy images of microneedle arrays. **(C)** 3D confocal reconstruction of a microneedle array. The FITC loaded tips are shown in green, while the sulphorhodamine B loaded PLGA μPs are in red.

The morphological features of the microneedles were investigated by using optical, scanning electron, and confocal microscopy (Figure 4).

#### Optical Microscopy

An optical stereomicroscope was employed to visualize the array and tips of the microneedles. The image shows (Figure 4A) a whole array of uniformly distributed 600 μm height microneedles with sharp tips.

#### Scanning Electron Microscopy

SEM imaging was used to visualize the morphology of freshly fabricated 600 μm height microneedles. The image (Figure 4B) shows that the final multi-compartmental microneedles clearly reflect the stamp cavity architecture with fine and sharp tips. The base diameter and the height of the microcones are 300 and 600 μm, respectively, in agreement with the design parameters.

#### Fluorescent Microscopy

Fluorescent imaging analyses were performed by means of confocal microscopy. The aim was to characterize the multi-compartmental structure of the microneedles using two different hydrophilic chromophores as reported in Figure 4C. The polymeric matrix was loaded with FITC, while the pores of the μPs were loaded with sulphorhodamine B. The 3D reconstructed image clearly shows that the PVP compartment (loaded with FITC) is located at the tip of the microneedles. On the other hand, as expected, the μPs (loaded with sulphorhodamine B) are located in the body of the microneedles.

### Image Analysis of Microneedles and Microparticle Slices

**Figure 5:**
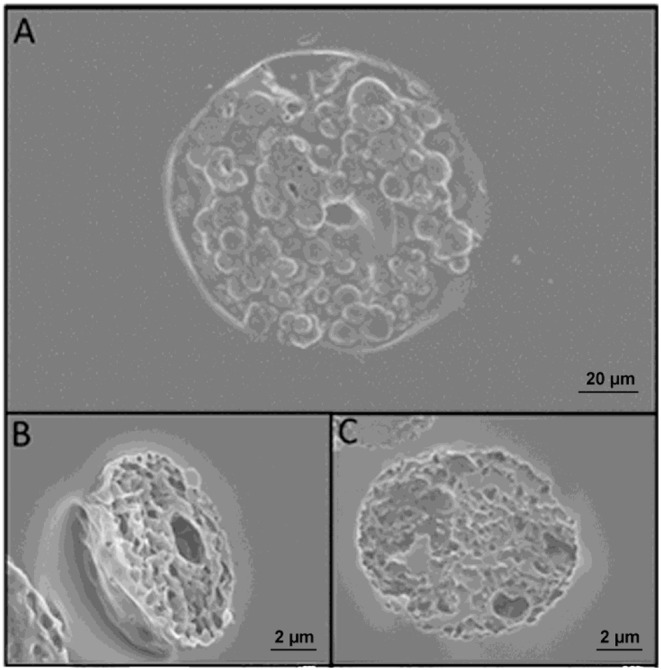
**(A)** SEM images of microneedles slices obtained with cryosectioning. **(B)** Slice of a microparticle contained in the microneedle after sintering. **(C)** Slice of a microparticle just prepared. A comparison of **(B,C)** reveals that sintering does not change the morphology of the microparticles.

Morphological analyses were performed using a field emission SEM on porous microneedles and μPs slices. Images of both multi-compartmental microneedles and μPs were acquired. Figure 5 shows that a similar porosity distribution can be observed on both samples, demonstrating that the microneedle production process does not significantly affect μPs morphology. This evidence suggests that in principle it is possible to import and exploit the extensive background of the PLGA μPs in terms of encapsulation and control of the kinetic release of the microneedles.

### *In vitro* Penetration Studies

**Figure 6:**
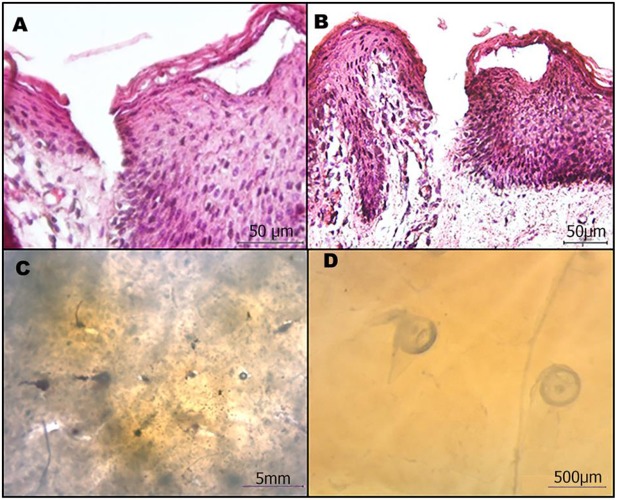
Microneedle patches efficiently penetrate the skin. **(A,B)** Cross-sectional images of the H/E stained skin after microneedle removal. **(C,D)** Stereomicroscope image of microneedles in the skin.

It is fundamental that microneedles are able to pierce the stratum corneum of the skin. To assess this aspect, an MPatch™ applicator system (see Materials and Methods), which is recommended for performing reproducible indentations, was used. Pig cadaver skin was used as a model for the indentation test, and a patch of microneedles as previously described was applied to it for 5 min. The effectiveness of the indentation was confirmed by the cross section of indented skin stained with H/E (Figure 6, upper line). In addition, the optical image also shows the microneedles in the skin upon indentation and medical tape removal (Figure 6, bottom line).

The H/E images clearly show that the microneedles reached the dermis, thus confirming the potential use for drug delivery. In fact, they penetrated all the layers of the epithelium and part of the papillary dermis.

### Enzyme Encapsulation in Microneedles

The encapsulation of catalyzers in biodegradable polymeric microneedles is a simple and effective method to deliver enzymes into human skin for dermatology and cosmetic treatments (Haj-Ahmad et al., 2015; Pezzella et al., 2017). The cosmetic sector is interested in enzymes that enhance the beauty of the skin or help the skin to defend itself against the ravages of the environment. However, since creams are not effective in the delivery of compounds with high molecular weight, molecules, such as proteins and invasive injection systems are typically used for enzyme dosage. Painless biodegradable polymeric microneedles therefore represent a promising tool.

In the present work, we loaded polymeric and multi-compartmental microneedles with recombinant laccase POXA1b belonging to the edible fungus Pleurotus ostreatus as a model enzyme (Pezzella et al., 2013, 2017). Laccases are an interesting group of multi copper enzymes, which have received much attention in several biotechnological processes (Lettera et al., 2016; Patel et al., 2019).

**Figure 7:**
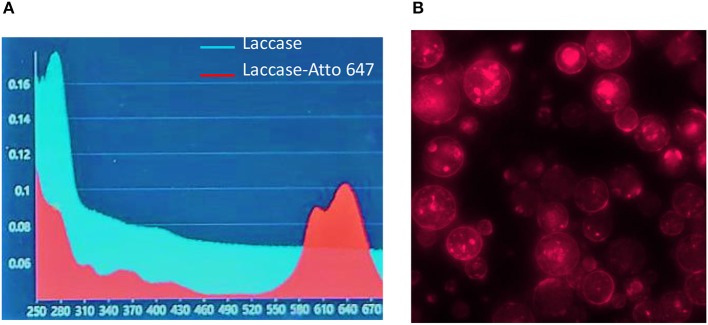
Laccase-Atto 647 μPs. **(A)** UV spectrum of Laccase (light blue line) and Laccase Atto647 (red line). **(B)** Fluorescence image of Laccase-Atto647 μPs.

**Figure 8:**
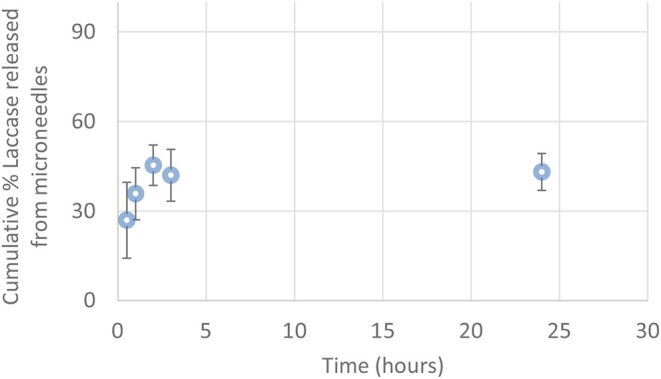
Cumulative percentage of Laccase-Atto647 released from μPs in the microneedles (0.5–24 h) (*n* = 3).

In order to evaluate the content of the enzyme encapsulated in μPs and its release in physiological conditions, we conjugated a dye (Atto647) to the Lysine side chain in the protein, as reported in the Materials and Methods. The correct conjugation was evaluated by UV, monitoring the presence of two shoulders at 600 and 643 nm that are typical of the dye and the signal at 280 nm related to the protein (Figure 7A). We obtained a perfect degree of loading (DOL) equivalent to 2. Thereafter, we showed, using a fluorescence technique, that this formulation was able to encapsulate a high content of protein equal to 77.5% ± 3.5. This data was in ageemento with fluorescence imaging (Figure 7B) which highlighted a high fluorescent enzyme signal. Thanks to the fluorescence assays we were able to calculate that μPs from microneedles released 43.1% ± 6.2 of the laccase over 24 h. This result means the microparticles did not lose their morphological structure after plasticization and were thus still able to release the drug in a time frame of hours (Figure 8).

**Figure 9:**
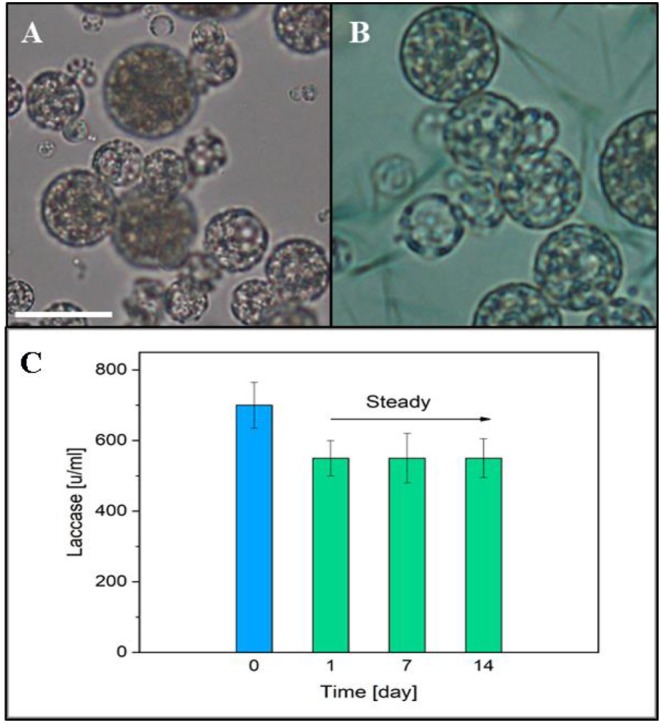
**(A)** Optical image of μPs slice. **(B)** Optical μPs slice with a solution of ABTS and buffer. Scale bar 10 μm. **(C)** Laccase activity in μPs up to 14 days after the production. The blue column represents the initial enzyme activity used for μPs preparation.

A laccase activity assay was used to monitor the presence and the preservation of laccase encapsulated in the PLGA μPs pores. A solution of ABTS and buffer was added onto the μPs slices in order to verify the enzyme activity. Figures 9A,B show the differences between the enzyme-loaded μPs slices in the presence and absence of ABTS, respectively. The ABTS solution highlights the enzyme activity, and the activity of the enzyme inside the μPs was confirmed by the presence of a green color located in the μPs pores due to the ABTS oxidation from the encapsulated enzyme itself. The activity of Laccase μPs was monitored for up to 14 days after production and after a slow reduction, registered in the first day, it was stable over time (Figure 9C).

### Enzyme Release and Activity in Skin Models

To check the enzyme activity, a preliminary test was performed on a 3 mm gelatin B substrate embedding the ABTS substrate. The results confirmed the activity of the encapsulated enzyme as reported in Figure S2.

**Figure 10:**
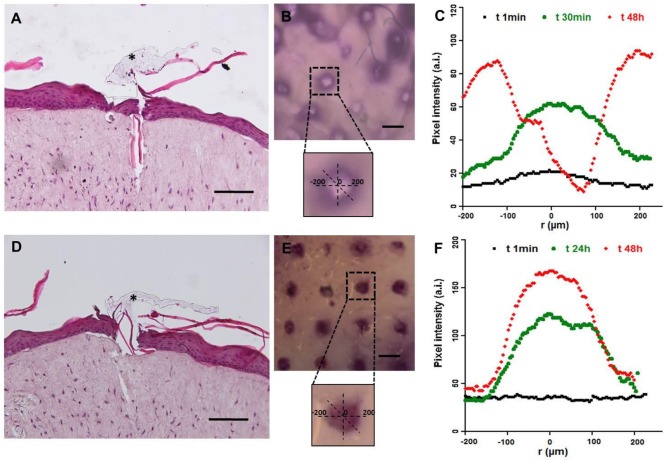
Functional test of microneedles for transdermal delivery of high molecular weight substances in a full-thickness human skin model. The pictures in **(A–C)** refer to microneedle configuration 1, while **(D–F)** refer to configuration 2. **(A,D)** Histological images of Endo-HSE 48 h after microneedle indentation to highlight the transdermal penetration; black asterisks indicate the PVP polymer remaining after medical tape removal (scale bar = 100 μm). **(B,E)** Stereomicroscopic images of Endo-HSE 48 h after indentation (scale bar = 500 μm). The inserts of the stereomicroscopic images are the schematic representation of the methods used to calculate the diffusive radius. **(C,F)** The graphs plot the pixel intensity at three time points correlated to the concentration of the ABTS oxidation product diffusing into ECM vs. the radius of the diffusion pattern.

Microneedles are designed to guarantee the reproducible transdermal indentation as well as the controlled and functional release of the encapsulated substance. To assess the microneedles' ability to satisfy such requirements, a human skin equivalent (Endo-HSE) was used as assay platform. Endo-HES is a functional and histological competent tissue that captures several fundamental features of the native human skin (Casale et al., 2016, 2018). In addition, in contrast to the use of pig skin, Endo-HSE is of human origin and can reproduce the sample's thickness as well as ECM compactness, thus avoiding the variability correlated to the age and region of the animal. Moreover, compared with other full thickness human skin models (Bellas et al., 2012; Carriel et al., 2012) that are formed with exogenous biopolymers, such as collagen and/or elastin, Endo-HSE is completely scaffold-free possessing a 1-mm thick dermis that is continuously assembled and remodeled by fibroblasts. The presence of such living and endogenous dermis may represent the best *in vitro* condition to perform transdermal transport studies. Histological images of Endo-HSE cross sections after the indentation highlighted that the microneedles were able to penetrate Endo-HSE, going through the epidermis and reaching a depth of ~250 μm for both microneedle configurations (Figures 10A,D).

The stereomicroscope images revealed the pattern of the microneedles' array and the ability of the enzyme loaded in each microneedle to react with the ABTS substrate. In fact, the ABTS oxidation product was blue and was detected easily. In the case of microneedle configuration 1, the ABTS oxidation occurred in a very short time since the enzyme loaded in the microneedle tip was rapidly available. In the case of microneedle configuration 2, the observation time was several hours because the laccase diffusion occurred upon μPs biodegradation.

Figures 10C,F plot the pixel intensity along the diffusion radius of the ABTS oxidation product for three time points. For configuration 1, diffusion began after 1 min and its radius was centered with the microneedle pattern for at least the first half hour, increasing for longer times as shown at 48 h. At 48 h, we observed an inversion of the bell apex indicating that no more enzymes were present in the microneedles, and, as a consequence, the ABTS oxidation product (dark pixels) was no longer present. The inversion of the bell apex was due to the accumulation of the ABTS oxidation product around the body of the microneedles (made of microparticles), which is located in the center of the pattern.

In configuration 2, the reaction product diffused more slowly than in configuration 1, and the concentration profile was flat at *t*_0_. The PLGA μPs adsorbed the ABTS, which reacts inside the microneedles with the active enzyme, as reported at 24 h in Figure 10F. At 48 h, we still observed an intense staining in relation to the indented region very close to the walls of the microneedles (Figure 10F) due to the localization and preservation of the enzyme within μPs and its slow release from μPs.

We stopped observations at 48 h because the ABTS soaked in the Endo-HSE was by then oxidized by the air present in the atmosphere thereby making the sample completely dark.

In conclusion, the experimental results showed that the activity of the enzyme was preserved after microneedle production in both encapsulation procedures. In addition, we demonstrated the more rapid diffusion of the encapsulated enzyme in configuration 1 compared to configuration 2 (Figures 10B,C,E,F). The release of the ABTS oxidation product through PLGA μPs occurred by means of a combined diffusion-degradation mechanism, which led to the prolonged release in the ECM. In fact, in configuration 2, the diffusion-controlled systems based on slow-biodegradable polymers were designed for the controlled release of the encapsulated substance. By encapsulating the enzyme in both compartments, therefore, we can combine a fast release with a prolonged release depending on the type of application required.

## Conclusions

In this work we have presented a novel, fast, and stamp-based procedure for the fabrication of microneedles.

First, the master production process was optimized by using a 2PP technique. In addition, the procedure to fabricate the microneedles was designed with an RT procedure that allows the use of thermo-sensitive biomolecules, such as laccase.

The tips of the microneedles are made of a biodegradable and biocompatible polymer, such as PVP, and the bodies of the microcones are made up of mild sintered PLGA microparticles. Both compartments can entrap therapeutic agents, leading to the production of a biodegradable, biocompatible and multi-compartmental system. The multi-compartmental feature enables the co-delivery of two molecules, along with a rapid and prolonged release of the cargo. This release was confirmed by the diffusion test. In fact, when the enzyme was incorporated into the hydrophilic matrix, there was an instantaneous release of the cargo, but when the enzyme was incorporated into the μPs, it was released over a prolonged period.

This method preserved the porous microstructure of the μPs, as testified by the morphological characterizations carried out, and the prolonged release of the cargo can be tuned by engineering the properties of the initial μPs.

In addition, due to the thin interconnecting layer between microneedles they can be integrated onto a flexible patch keeping the flexibility of the entire patch to improve the conformal contact with the skin and to facilitate the implantation of the microneedles upon indentation, as revealed during our *in vitro* tests.

The overall fabrication procedure is faster compared to other stamp-based techniques, this is fundamental for the possible future marketing of such a tool.

## Data Availability Statement

All datasets generated for this study are included in the article/Supplementary Material.

## Author Contributions

RV and MB conceived the experiments and the plasticizing setup, analyzed the main results. MB optimized the experimental protocol for the production of microneedles. RJ provided help in microneedles and microparticles preparation and bted to the layout of the images and TOC. MP contributed to the activity tests. CD carried out the Laccase-Atto 647 conjugation, enzyme encapsulation in PLGA microparticles together with morphological analysis and release tests. CC, GI, and FU contributed to the design and realization of the *in vitro* skin model. CC performed the characterization of the indentation tests. VL provided the enzyme and contributed to the activity tests. MB, RV, MP, and FP wrote the main manuscript text. PN provided the theoretical support and reviewed the manuscript.

### Conflict of Interest

VL was employed by company Biopox Srl, Naples. The remaining authors declare that the research was conducted in the absence of any commercial or financial relationships that could be construed as a potential conflict of interest.
