# Supplementary material for: Non-invasive Production of Multi-Compartmental Biodegradable Polymer Microneedles for Controlled Intradermal Drug Release of Labile Molecules
Source: Front Bioeng Biotechnol. 2019 Nov 8;7:296. doi: 10.3389/fbioe.2019.00296 (PMC6856554; doi:10.3389/fbioe.2019.00296)
Supplement: Supplementary file 1 [file Image_1.pdf]

Non-invasive production of multi-compartmental biodegradable polymer microneedles for controlled intradermal drug release of labile molecules

Mario Battisti,[1,†], Raffaele Vecchione[1,†,], Costantino Casale[2], Fabrizio A Pennacchio[1], Vincenzo Lettera[4], Rezvan Jamaledin[1], Martina Profeta[1], Concetta Di Natale[1], Giorgia Imparato[1], Francesco Urciuolo[2,3], Paolo Antonio Netti[1,2,3]*

[1]Center for Advanced Biomaterials for Health Care (CABHC), Istituto Italiano di Tecnologia, Largo Barsanti e Matteucci 53, Naples, Italy

[2]Interdisciplinary Research Centre on Biomaterials (CRIB), University of Naples Federico II, P.le Tecchio 80, Naples, 80125, Italy

[3]Department of Chemical Materials and Industrial Production (DICMAPI), University of Naples Federico II, P.le Tecchio 80, Naples, 80125, Italy

[4]Biopox Srl, Salita Arenella 9, Naples, Italy

* Corresponding Author:

E-mail address: raffaele.vecchione@iit.it;

[†]Mario Battisti and Raffaele Vecchione contributed equally to this work

Supporting Info

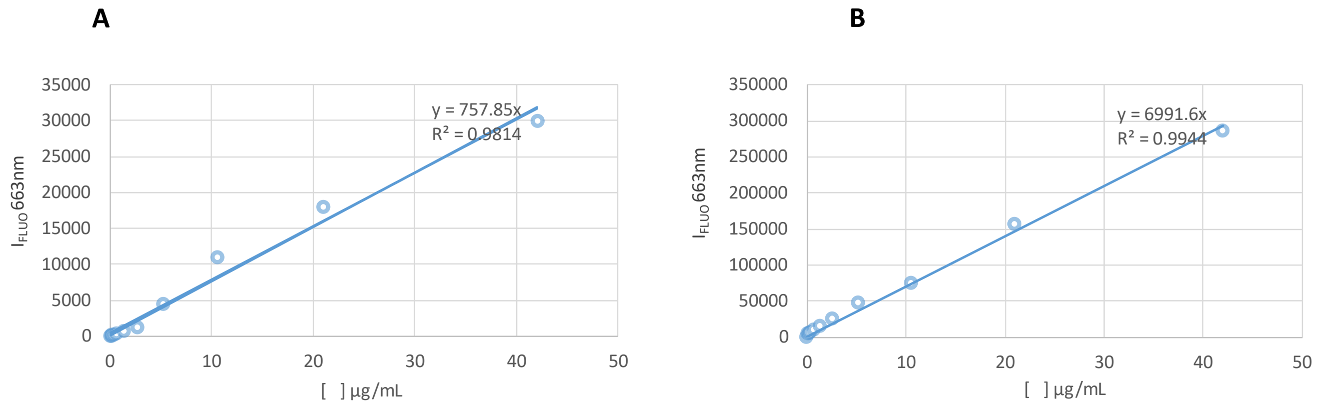

Figure S1 Titration curve of Laccase-Atto647 in A) 50% DMSO/0.1M NaOH/0.5%SDS;B) PBS pH 7.1.

Release and activity of the enzyme in a gelatin model

Figure S2 shows the effect of microneedles in the gelatin after indentation with an MPatch™ applicator system. The first column refers to the microneedles loaded with the enzyme-loaded tips. The central column refers to the microneedles with both the tip and the µPs loaded with the enzyme. The third column refers to the microneedles with empty tips and enzyme-loaded µPs. This analysis shows the different time scale release of the encapsulated enzyme based on the compartment of the microneedles. In fact, when the enzyme was present in the tip, it was already possible to observe the oxidation of the enzyme due to the ABTS encapsulated in the gelatin after 1 h, while when the enzyme was encapsulated in the µPs, this phenomena was only clearly observable after 48 h.

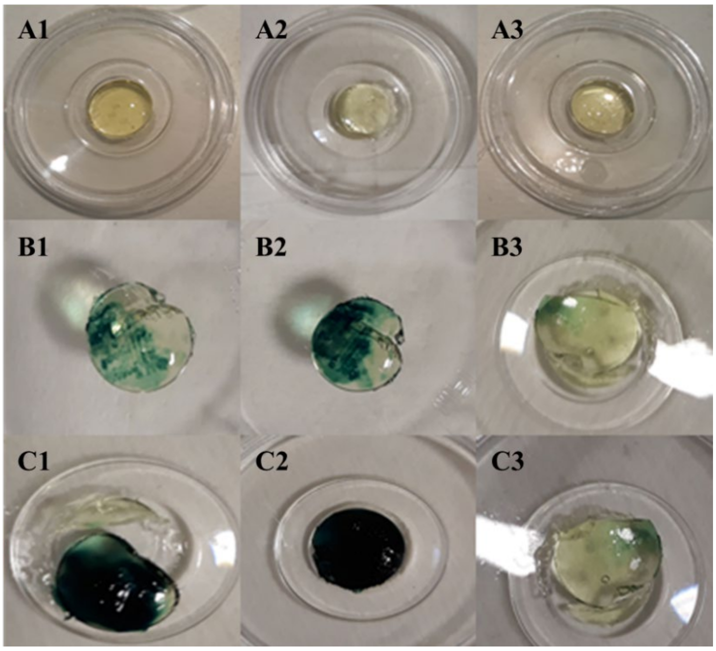

Figure S2 (A1) (A2) (A3) Gelatin substrate with a PDMS layer coating before indentation. (B1) (B2) (B3) 1 h after the indentation; (C1) (C2) (C3) 24 h after the indentation. The first column refers to the enzyme in the PVP tip, the second to the enzyme in both compartments, and the third to the enzyme loaded in the PLGA μPs.
